# Graphene-Driven Formation of Ferromagnetic Metallic Cobalt Nanoparticles

**DOI:** 10.3390/nano16010041

**Published:** 2025-12-28

**Authors:** Salim Al-Kamiyani, Mohammed Al Bahri, Tariq Mohiuddin, Eduardo Saavedra, Al Maha Al Habsi

**Affiliations:** 1 Department of Basic and Applied Sciences, A’Sharqiyah University, P.O. Box 42, Ibra 400, Oman; 2 Physics Department, College of Science, Sultan Qaboos University, Al-Khoudh, Muscat 123, Oman; 3 Department of Physics, University of Santiago of Chile, Víctor Jara Avenue 3493, Santiago 9170124, Chile

**Keywords:** graphene, metallic cobalt nanoparticles, crystallographic structure, magnetic behavior and electronic band structure

## Abstract

This work demonstrates the synthesis of ferromagnetic metallic cobalt nanoparticles embedded in a graphene framework through a graphene-assisted carbothermal reduction process. Cobalt oxide (Co_3_O_4_) was employed as the starting material, with graphene nanopowder functioning simultaneously as the reducing medium and structural scaffold. Thermal treatment at 850 °C under an argon atmosphere triggered the phase transformation. X-ray diffraction (XRD) confirmed the successful conversion of cobalt oxide into face-centered cubic (FCC) metallic cobalt. The graphene network not only accelerated the reduction reaction but also ensured the homogeneous distribution of cobalt nanoparticles within the matrix. Magnetic measurements using vibrating sample magnetometry (VSM) revealed a substantial improvement in ferromagnetic behavior: the graphene-mediated samples reached a saturation magnetization (*M_s_*) of approximately 130 emu/g, compared to the nearly non-magnetic response of cobalt oxide annealed under the same conditions without graphene. Collectively, the structural, compositional, and magnetic results highlight graphene’s critical role in driving the formation of metallic cobalt nanoparticles with enhanced ferromagnetism, emphasizing their promise for use in magnetic storage, sensing, and spintronic applications. We anticipate that this study will inspire further research into the dual functionality of graphene, serving as both a reductive agent for metal oxides and a supportive matrix for nanoparticles, toward enhancing the structural integrity and functional properties of graphene-based metal nanocomposite materials.

## 1. Introduction

Cobalt-based nanomaterials have attracted considerable attention owing to their strong magnetic behavior and diverse applications in catalysis, magnetic data storage, sensing devices, and biomedical systems [1]. These functionalities are primarily associated with metallic cobalt or engineered cobalt–carbon composites, whereas cobalt oxides generally display weak or negligible magnetism at room temperature because of their insulating nature and antiferromagnetic ordering [2].

Graphene, recognized for its exceptional electrical conductivity, thermal stability, and large specific surface area, has been extensively employed as both a support matrix and a reductive medium for the transformation of metal oxides [1]. Previous research has reported the fabrication of cobalt or cobalt oxide nanostructures on graphene through hydrothermal [3], solvothermal [4], and deposition–precipitation routes [5], leading to improvements in stability, catalytic efficiency, and, in some cases, emergent magnetic properties [1]. For instance, Singh et al. synthesized shape-controlled Co nanoparticles on reduced graphene oxide (rGO), demonstrating size-dependent transitions between ferromagnetic and superparamagnetic responses [6]. This response is also sensitive to the crystal structure. While metallic cobalt nanoparticles typically adopt a cubic spinel-like structure, glass-like phases have also been reported, where the XRD pattern displays only a single broad peak [7].

Despite these advancements, the direct carbothermal reduction of cobalt oxides (Co_2_O_3_ or Co_3_O_4_) into ferromagnetic metallic FCC cobalt nanoparticles embedded within graphene remains insufficiently explored. Conventional carbothermal reduction typically relies on graphite or coal as a carbon source and requires high processing temperatures above 1000 °C [4,8]. Although graphene oxide has been utilized to reduce Co_3_O_4_ into cobalt carbide (Co_2_C) [9], no study to date has shown complete and direct conversion to metallic cobalt within a graphene scaffold, integrating both structural transformation and enhanced magnetic behavior.

Moreover, there has been limited effort to correlate graphene-driven reduction with simultaneous structural and magnetic validation using X-ray diffraction (XRD), band structure analysis, and vibrating sample magnetometry (VSM). Understanding how graphene mediates changes in cobalt’s crystal structure, electronic band configuration, spin polarization, and ferromagnetism under thermal treatment remains a critical knowledge gap.

To address this, the present study pursues three main objectives:

- Demonstrating the graphene-assisted carbothermal reduction of cobalt oxide into metallic FCC cobalt (Co) nanoparticles embedded within graphene at 850 °C.
- Establishing a direct one-step pathway for reducing Co_3_O_4_ to metallic cobalt while achieving enhanced ferromagnetism.
- Providing detailed structural (XRD), high-resolution XPS, magnetic (VSM), and electronic band structure analyses to clarify the role of graphene in enabling phase transformation and magnetic improvement.

This work presents a simple and efficient one-step graphene-assisted method that drives the reduction of cobalt oxide to crystalline metallic FCC cobalt while enhancing ferromagnetism, demonstrating graphene’s dual role as both a reductant and a stabilizing medium for the cobalt nanoparticles. These findings, consistent with earlier reports on Co–rGO systems [10,11], highlight new opportunities for designing graphene-based metal nanocomposites and underscore graphene’s pivotal contribution to advancing multifunctional materials for magnetic devices, electronics, and energy technologies.

## 2. Experimental Procedure

Four sets of samples were prepared to investigate the role of graphene in the thermal reduction of cobalt oxide (Co_3_O_4_) and the associated structural and magnetic changes; an annealing temperature of 850 °C was used since graphene develops a high density of structural defects, which can significantly influence its chemical reactivity and interfacial behavior [12].

Sample 1 (Pristine Graphene): Commercial graphene powder supplied by CiCarbo (Celtig LLC, Knoxville, TN, USA) Graphene was employed for this purpose.

Sample 2 (Pristine Co_3_O_4_): Commercial Co_3_O_4_ powder was purchased from GetNanoMaterials (GNM) (Saint-Cannat, France) and used without further treatment. This sample served as the reference material for comparison.

Sample 3 (Thermally Treated Co_3_O_4_ without Graphene): To assess the thermal effect in the absence of carbon, a portion of the same Co_3_O_4_ powder was annealed at 850 °C for 2 h under a continuous argon flow. This inert atmosphere prevented oxidation during high-temperature exposure.

**Figure 1:**
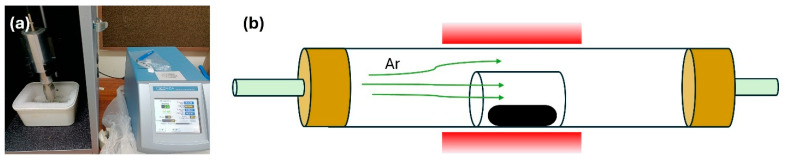
(**a**) Probe sonication unit. (**b**) Schematic illustration of the horizontal tube furnace.

Sample 4 (Graphene-Assisted Co–Graphene Composite): For the graphene-assisted sample, Co_3_O_4_ was blended with graphene nanopowder at a weight ratio of 4:1. The mixture was dispersed in deionized water and sonicated for 30 min using a Qsonica Q700W (Connecticut, CT, USA) ultrasonic probe to enhance dispersion and reduce agglomeration (Figure 1a). The resulting suspension was filtered and dried at room temperature. The dried composite was subsequently annealed at 850 °C under argon in a horizontal tube furnace (Figure 1b), following the same thermal profile as Sample 2.

## 3. Results and Discussion

### 3.1. Material Surface Morphology Characterization

**Figure 2:**
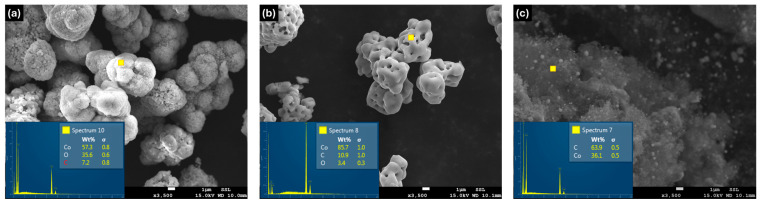
SEM images of (**a**) pristine Co_3_O_4_ before treatment, (**b**) Co_3_O_4_ annealed in argon ambient at 850 °C, and (**c**) Co_3_O_4_ annealed in argon ambient in presence of graphene at 850 °C.

Scanning Electron Microscopy (SEM) was carried out using a JEOL JSM-7800F00 (JEOL Ltd., Tokyo, Japan) operated at 15 kV to investigate the surface morphology of the prepared samples. The SEM image of the pristine Co_3_O_4_ powder (Figure 2a) shows cobalt oxide present as microscale particles. The elemental composition shows its approximate surface oxide ratio in the Energy-Dispersive Spectroscopy (EDS) spectrum. Following probe sonication and subsequent annealing, distinct morphological modifications are observed (Figure 2b). In contrast, the graphene-assisted sample clearly exhibits the formation of metallic cobalt nanoparticles uniformly embedded within the graphene matrix (Figure 2c).

### 3.2. X-Ray Diffraction (XRD) Analysis

**Figure 3:**
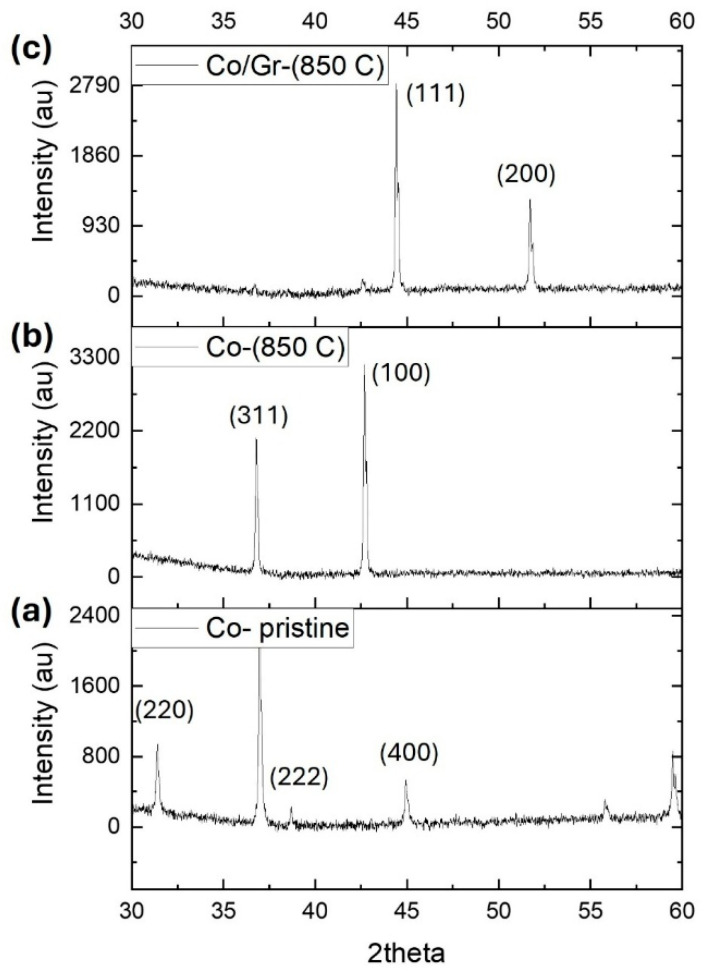
XRD pattern of (**a**) pristine Co_3_O_4_ before treatment, (**b**) Co_3_O_4_ annealed in argon ambient at 850 °C, and (**c**) Co_3_O_4_ annealed in argon ambient in presence of graphene at 850 °C.

X-ray diffraction (XRD) measurements were carried out on a PANalyticaldiffractometer (Model X’Pert Pro, USA) using Cu Kα radiation (λ = 1.5406 Å) to analyze the structural characteristics of the samples. The pristine Co_3_O_4_ powder displayed the typical diffraction peaks of cobalt oxide, confirming its initial crystalline phase (Figure 3a). After annealing at 850 °C without graphene, notable alterations in the diffraction pattern were observed (Figure 3b). A newly emerged peak indexed to the (100) plane signaled a phase transformation and a substantial modification in the lattice structure.

In contrast, the XRD pattern of the graphene-assisted sample (Figure 3c) showed distinct diffraction peaks near 44.2° and 51.5°, corresponding to the (111) and (200) planes of face-centered cubic (FCC) metallic cobalt, respectively. This confirms the successful reduction of Co_3_O_4_ into metallic cobalt and highlights the role of graphene in directing the phase transformation. The Debye–Scherrer equation is used to estimate the crystallite size of a nanocrystalline material from an X-ray diffraction (XRD) pattern (Figure 3c). The result shows an average particle size of 46.2 nm.

### 3.3. Crystallographic Structure

**Figure 4:**
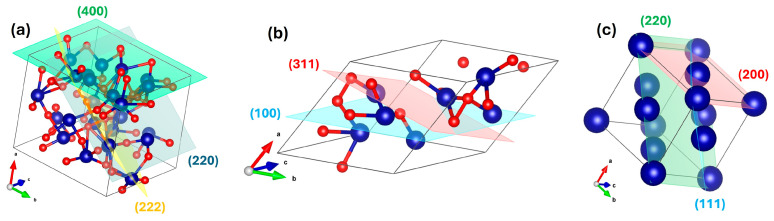
Crystallographic visual representation of (**a**) pristine Co_3_O_4_ before treatment, (**b**) Co_3_O_4_ annealed in argon ambient at 850 °C, and (**c**) Co_3_O_4_ annealed in argon ambient in presence of graphene at 850 °C.

To evaluate the influence of heat treatment and graphene on the crystal structure, XRD refinement was performed on all three samples using the Materials Analysis Using Diffraction (MAUD) software (Ver 2.9995, 2025) [13]. The pristine Co_3_O_4_ sample was refined with the standard crystallographic information file (CIF, ID: mp-18748) [14]. The refinement showed strong agreement with experimental data, yielding a weighted profile R-factor (Rwp) of 3.33%. Since values below 10% are typically considered reliable, this indicates a good fit. The refined structure corresponded to a spinel structure with space group H-M (Hermann–Mauguin). Following the structural refinement, a crystallographic information file (CIF) was generated from the refinement results using MAUD and subsequently visualized with the Visualization for Electronic and Structural Analysis (VESTA) software, version 3.4.7 [15], as illustrated in Figure 4a.

For the annealed sample without graphene (Figure 4b), refinement yielded an Rwp of ~2.88%. The analysis revealed a phase transformation to a face-centered cubic (FCC) lattice with diamond glide symmetry, assigned to space group Fd-3m (227), characteristic of spinel-like or diamond-related phases.

By contrast, the graphene-assisted sample exhibited an even lower Rwp of ~2.48%, confirming a highly symmetric FCC lattice belonging to space group Fm-3m (225), which is typical of metallic cobalt. The refined structure was also visualized in VESTA (Figure 4c). To validate the structural model, the interplanar spacing (d-spacing) was calculated and found to be consistent with the refined lattice parameters. This sample has a (111) peak at 2θ=44.2°, with $hkl⟹h^{2}+k^{2}+l^{2}=1+1+1=3$. For Cu Kα radiation λ = 1.5406 Å,

$$d=\frac{1.5406}{2sin(22.1)}=2.05Å⟹a=2.05Å\times \sqrt{3}=3.55Å$$

These results clearly demonstrate that the presence of graphene enhances the reduction process, enabling the efficient conversion of cobalt oxide into metallic cobalt under annealing conditions. The refined lattice parameters for all samples are summarized in Table 1.

### 3.4. High-Resolution XPS Analysis

High-resolution X-ray photoelectron spectroscopy (XPS) was conducted (Scienta Omicron, Germany) to investigate the surface chemical states of the samples, including pristine graphene (Gr), Co_3_O_4_, Co_3_O_4_ annealed at 850 °C (Co–850 °C), and the graphene-assisted composite (Co/Gr–850 °C). To compensate for surface charging during measurement, the system utilized a built-in low-energy electron flood gun. All spectra were calibrated to the C 1s peak at 284.6 eV, and the Shirley background was subtracted for more accurate comparison of elemental composition and electronic states.

**Figure 5:**
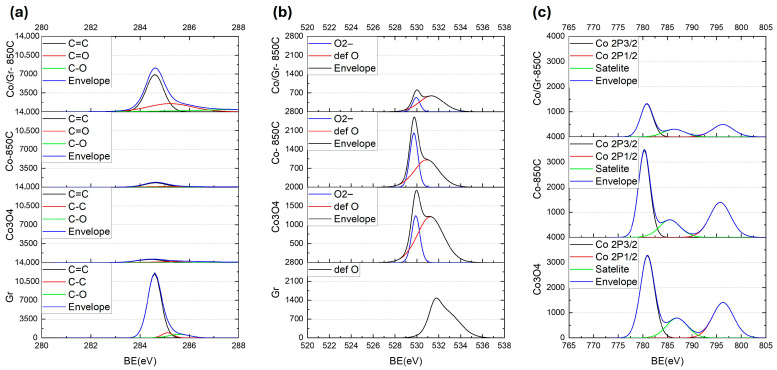
High-resolution XPS peaks of (**a**) C 1s, (**b**) O1s of C 1s, (**c**) Co 2p_3/2_ and 2p_1/2_ spectra.

Figure 5a shows the C 1s spectra. Pristine graphene exhibits a dominant sp^2^ C=C peak at 284.6 eV, accompanied by minor contributions from C–C and C–O functional groups. This characteristic feature remains clearly observable, though with reduced intensity, in the Co/Gr–850 °C composite, confirming that the thermal treatment leads to partial carbon loss while preserving the graphene framework. The presence of residual oxygen-containing species (C–O and C=O) in the Co/Gr–850 °C sample indicates partial surface oxidation of graphene or defect sites. In contrast, the Co–850 °C and Co_3_O_4_ samples display significantly weaker C 1s signals, as expected due to the absence of graphene.

The O 1s spectra shown in Figure 5b reveal key insights into oxygen environments. The pristine Co_3_O_4_ sample displays two well-resolved components: a main peak centered at 529.9 eV with FWHM of 0.89 eV, attributed to lattice oxygen (O^2−^), and a broader secondary peak near 531.2 eV (FWHM = 2.82 eV), assigned to defect-related oxygen (def O), oxygen vacancies, or adsorbed oxygen species. These features are characteristic of the spinel structure with mixed-valence Co^2+^/Co^3+^. Upon thermal treatment without graphene (Co–850 °C), the intensity of the lattice oxygen signal (O^2−^) remains prominent, though the defect oxygen peak decreases in intensity, indicating surface reduction. In contrast, the graphene-assisted composite (Co/Gr–850 °C) shows a significantly suppressed lattice oxygen signal (O^2−^), confirming that graphene facilitates substantial reduction of cobalt oxide during annealing. This is consistent with the transformation from Co_3_O_4_ to metallic cobalt. The presence of a residual defect oxygen peak may arise from graphene edge functionalities or trapped oxygen species on graphene.

Figure 5c displays the high-resolution Co 2p spectra. In pristine Co_3_O_4_, two main peaks are observed at 780.9 eV (Co 2p_3/2_) with FWHM of 3.4 eV and 796.3 eV (Co 2p_1/2_), alongside a strong satellite feature at about 786 eV, a signature of Co^2+^ in the Co_3_O_4_ spinel phase. These features are also retained in the Co–850 °C sample, though with reduced intensity of the satellite, reflecting incomplete reduction. However, in the graphene-assisted Co/Gr–850 °C sample, the Co 2p spectrum exhibits diminished satellite intensity and Co 2p_3/2_ peak shape with FWHM of 2.9 eV. The weakening of the satellite peak near 786 eV suggests a substantial loss of Co^2+^ species and the emergence of metallic Co, consistent with the reduction promoted by graphene. The residual minor satellite component may be attributed to remaining oxygen defects or trapped oxygen on graphene, likely associated with defective graphene sites rather than cobalt oxide remnants.

### 3.5. Magnetic Behavior

**Figure 6:**
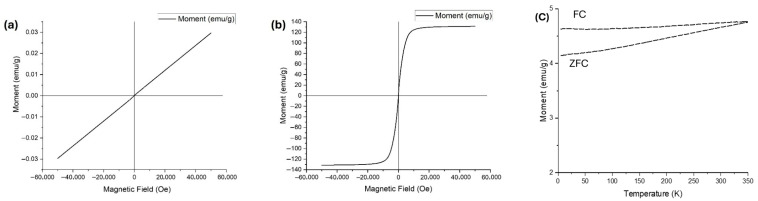
VSM curves for cobalt oxide (**a**) annealed in argon ambient at 850 °C, and (**b**) annealed in argon ambient in presence of graphene at 850 °C, (**c**) ZFC and FC magnetization curves of sample annealed in argon ambient in presence of graphene at 850 °C.

Magnetic measurements were analyzed with a Quantum Design PPMS DynaCool magnetometer (Quantum Design Inc, CA, USA. The magnetic behavior of cobalt oxide samples annealed at 850 °C with graphene and without graphene is illustrated in the Vibrating Sample Magnetometer (VSM) hysteresis curves presented in Figure 6. The sample annealed without graphene (Figure 6a) exhibits a linear magnetization response with negligible hysteresis, indicating predominantly paramagnetic behavior. The moment remains near zero across the entire applied magnetic field range, consistent with the expected weak or non-magnetic characteristics of cobalt oxide (Co_3_O_4_) after temperature treatment [16]. The slight slope observed reflects a very low magnetic susceptibility, further confirming the limited room-temperature magnetism of cobalt oxide phases.

In sharp contrast, the graphene-assisted sample (Figure 6b) displayed a pronounced ferromagnetic hysteresis loop, with rapid saturation magnetization normalized to the total mass reaching ~±130 emu/g and coercivity (*H_c_*) of 5.23 Oe. This ferromagnetic signal highlights the effective alignment of magnetic moments under the applied field, confirming the successful formation of metallic cobalt [17]. To confirm the magnetic behavior of the annealed sample in the presence of graphene, ZFC–FC magnetization curves (Figure 6c) were recorded over the temperature range of 5–350 K. Both ZFC and FC curves display magnetic moments exceeding 4 emu/g, suggesting a strong intrinsic magnetic response. The smooth and continuous increase in the ZFC curve, accompanied by a clear and persistent divergence from the FC curve, indicates irreversible magnetic behavior. The absence of a distinct blocking temperature further supports the classification of the material as ferromagnetic.

These findings emphasize graphene’s pivotal role in driving the phase transformation and significantly enhancing magnetic performance. The robust ferromagnetism observed in the graphene-assisted composites positions them as promising candidates for applications in flexible magnetic sensors [18], data storage technologies [19], and electromagnetic devices [20], where strong, tunable magnetic properties are essential.

### 3.6. Role of Graphene During Annealing

The integration of graphene into cobalt oxide significantly impacts the structural and magnetic characteristics of the resulting cobalt nanoparticles. The enhanced magnetic performance is primarily attributed to the efficient transformation of cobalt oxide into metallic cobalt [21], driven by carbon diffusion from graphene into the oxide lattice during annealing at 850 °C. X-ray diffraction (XRD) confirmed the formation of a face-centered cubic (FCC) metallic cobalt phase, a structure well recognized for its high saturation magnetization and excellent structural stability [22].

Beyond enabling phase conversion, graphene also provides a dual function: it acts as a physical support that ensures uniform dispersion of nanoparticles and as a reducing medium that creates the favorable environment necessary for carbothermal reduction. In this role, graphene serves as both an active carbon source and a reductant, effectively promoting the conversion of Co_3_O_4_ to metallic cobalt under high-temperature treatment.

The process can be simplified by the following reaction:

1. Decomposition of Co_3_O_4_ to CoO.
  $${Co}_{3}O_{4}\left(s\right)⟶3CoO\left(s\right)+\frac{1}{2}O_{2}(g)$$
2. Reduction of CoO by graphene (C) to metallic cobalt.
  $$CoO\left(s\right)+C(s)⟶Co\left(s\right)+CO$$

The overall reaction can be represented as:

$${Co}_{3}O_{4}\left(s\right)+4C(s)⟶3Co\left(s\right)+4CO(g)$$

In this process, carbon originating from graphene drives the reduction of cobalt oxide into metallic cobalt. The two-dimensional structure of graphene, combined with its large surface area and delocalized π-electron network, enhances its functional role within the composite. These characteristics enable efficient electronic interaction with cobalt nanoparticles, thereby improving catalytic performance. Additionally, the oxygen-containing groups present in the graphene (as shown in Figure 5) can coordinate with Co^2+^ ions released from the cobalt oxide precursor. This interaction promotes localized nucleation, leading to the uniform growth and dispersion of cobalt nanocrystals across the graphene matrix. Graphene’s strong adsorption capacity and surface reactivity further stabilize the nanoparticles and suppress agglomeration in the final material.

During annealing, graphene fulfills two critical functions: it provides the carbon necessary for carbothermal reduction, and it acts as a structural template that governs the formation and stabilization of cobalt nanoparticles. This approach goes beyond simple material mixing, representing a rationally engineered strategy to design highly stable, structurally optimized cobalt–graphene nanocomposites with enhanced magnetic, catalytic, and electronic performance. Adjusting graphene’s intrinsic properties—such as sheet size, degree of reduction, and functional group density—makes it possible to precisely tune the composite’s architecture as well as its electronic and magnetic characteristics, addressing persistent challenges like catalyst deactivation and nanoparticle instability.

### 3.7. DFT-Based Explanation of Magnetic Behavior in Co_3_O_4_ and Graphene-Driven Metallic Cobalt

To interpret the experimental findings, density functional theory (DFT) simulations were carried out on two representative phases, using the lattice parameters from XRD measurements: (i) the pristine Co_3_O_4_ spinel structure and (ii) the FCC cobalt (Co) structure obtained via graphene-assisted reduction. The aim was to correlate the observed magnetic behavior with the underlying differences in crystal symmetry and electronic band structure. Calculations were performed using VASP 6.3.0 within the generalized gradient approximation (GGA) and the Perdew–Burke–Ernzerhof (PBE) exchange–correlation functional. Projector augmented wave (PAW) pseudopotentials and a plane-wave cutoff of 520 eV were used. The structural parameters from experimental XRD refinement were directly incorporated to ensure realistic modeling. Strong on-site Coulomb interactions within the Co 3d orbitals were applied using the DFT + U approach with an effective U value of 3.3 eV. All calculations were conducted on a 6 × 6 × 6 Γ-centered k-point mesh, with energy and force convergence thresholds of 10^−6^ eV and 0.01 eV·Å^−1^, respectively. The initial magnetic moment of 2.5 μ_B_ per atom was applied to capture its intrinsic magnetic nature. Band structures were calculated along the high-symmetry path Γ–M–K–Γ–A.

**Figure 7:**
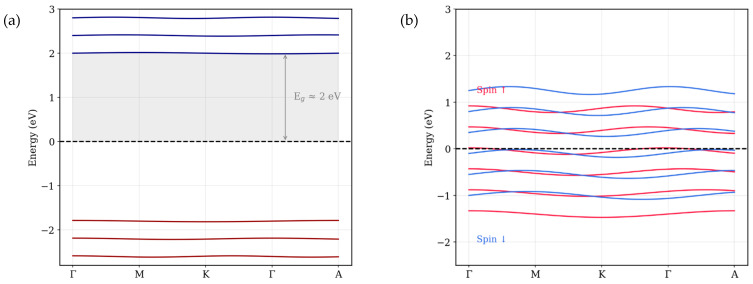
Simulated band structures of (**a**) pristine Co_3_O_4_ before treatment and (**b**) graphene-driven cobalt.

As shown in Figure 7a, the band structure of Co_3_O_4_ exhibits a band gap of about 2 eV with no evident spin splitting, consistent with the absence of long-range magnetic order and weak inter-site coupling. This result aligns closely with earlier first-principles studies, such as those by Singh et al. [23] and Chen et al. [24], who reported indirect band gaps ranging from 1.6 to 2.2 eV. These electronic features correlate well with the XPS spectra (Figure 5), which display satellite peaks near 786 eV, indicative of the presence of divalent cobalt (Co^2+^) ions within a material. The negligible magnetization and coercivity observed in VSM measurements further support the weakly magnetic nature of this sample [25].

In contrast, the graphene-assisted FCC cobalt sample exhibits a markedly different electronic signature. As shown in Figure 7b, significant spin splitting appears near the Fermi level: the spin-up bands shift below the Fermi level, while spin-down bands cross it—an electronic hallmark of ferromagnetic metals. These DFT results strongly agree with the VSM measurements, confirming ferromagnetic behavior. Importantly, the XPS 2p orbitals spectra (Figure 5c) for this sample show no significant satellite peaks, confirming the full reduction of cobalt into its metallic state. Additionally, the presence of spin asymmetry supports the refined FCC symmetry seen in XRD patterns (Figure 4), validating the formation of metallic Co(111) and (200) crystal planes.

Together, these theoretical and experimental insights confirm that graphene-assisted annealing drives a transformation from a weakly magnetic, insulating oxide into a metallic, spin-polarized ferromagnetic phase, with graphene serving as both a chemical reductant and a stabilizer for the resulting FCC cobalt nanostructure.

## 4. Conclusions

This work reports the successful synthesis of ferromagnetic metallic cobalt nanoparticles embedded in a graphene matrix via carbothermal reduction of cobalt oxide at 850 °C under argon. Structural and magnetic analyses (XRD and VSM) confirmed the graphene-assisted transformation, with the composite exhibiting a clear ferromagnetic hysteresis loop and a high saturation magnetization of ~130 emu/g, in sharp contrast to the negligible magnetism of samples annealed without graphene.

Density functional theory (DFT) provided further insight, revealing the transition from the insulating band structure of pristine Co_3_O_4_ to a spin-polarized metallic state in FCC cobalt, consistent with experimental observations.

These results highlight graphene’s critical role as both a reductant and structural stabilizer, enabling efficient phase conversion and enhanced magnetic properties. The scalable synthesis approach opens opportunities for designing cobalt–graphene nanocomposites with tunable ferromagnetism for advanced applications in magnetic sensors, electromagnetic devices, and biomedical technologies.

## Figures and Tables

**Table 1:** Lattice parameters for each sample after XRD refinement using MAUD software.

Sample	Unit-Cell Volume (Å^3^)	a (Å)	b (Å)	c (Å)	Alpha (α)°	Beta (β)°	Gamma (γ)°
Pristine COBALT OXIDE (Co_3_O_4_)	568.64	6.94	9.21	9.26	97.05	78.75	100.58
Co_3_O_4_/Argon 850C	164.02	6.10	6.32	6.18	57.53	59.05	60.15
Co_3_O_4_/Argon/Graphene 850C	044.56	3.55	3.55	3.55	90.00	90.00	90.00

## Data Availability

The original contributions presented in this study are included in the article. Further inquiries can be directed to the corresponding author.
